# Cultivation-Independent Methods Reveal Differences among Bacterial Gut Microbiota in Triatomine Vectors of Chagas Disease

**DOI:** 10.1371/journal.pntd.0001631

**Published:** 2012-05-01

**Authors:** Fabio Faria da Mota, Lourena Pinheiro Marinho, Carlos José de Carvalho Moreira, Marli Maria Lima, Cícero Brasileiro Mello, Eloi Souza Garcia, Nicolas Carels, Patricia Azambuja

**Affiliations:** 1 Laboratório de Biologia Computacional e Sistemas, IOC, FIOCRUZ, Rio de Janeiro, Brazil; 2 Laboratório de Bioquímica e Fisiologia de Insetos, IOC, FIOCRUZ, Rio de Janeiro, Brazil; 3 Laboratório de Doenças Parasitárias, IOC, FIOCRUZ, Rio de Janeiro, Brazil; 4 Laboratório de Ecoepidemiologia da Doença de Chagas, IOC, FIOCRUZ, Rio de Janeiro, Brazil; 5 Laboratório de Biologia de Insetos, UFF, Rio de Janeiro, Brazil; 6 Laboratório de Genômica Funcional e Bioinformática, IOC, FIOCRUZ, Rio de Janeiro, Brazil; SBRI, United States of America

## Abstract

**Background:**

Chagas disease is a trypanosomiasis whose agent is the protozoan parasite *Trypanosoma cruzi*, which is transmitted to humans by hematophagous bugs known as triatomines. Even though insecticide treatments allow effective control of these bugs in most Latin American countries where Chagas disease is endemic, the disease still affects a large proportion of the population of South America. The features of the disease in humans have been extensively studied, and the genome of the parasite has been sequenced, but no effective drug is yet available to treat Chagas disease. The digestive tract of the insect vectors in which *T. cruzi* develops has been much less well investigated than blood from its human hosts and constitutes a dynamic environment with very different conditions. Thus, we investigated the composition of the predominant bacterial species of the microbiota in insect vectors from *Rhodnius*, *Triatoma*, *Panstrongylus* and *Dipetalogaster* genera.

**Methodology/Principal Findings:**

Microbiota of triatomine guts were investigated using cultivation-independent methods, i.e., phylogenetic analysis of 16s rDNA using denaturing gradient gel electrophoresis (DGGE) and cloned-based sequencing. The Chao index showed that the diversity of bacterial species in triatomine guts is low, comprising fewer than 20 predominant species, and that these species vary between insect species. The analyses showed that *Serratia* predominates in *Rhodnius*, *Arsenophonus* predominates in *Triatoma* and *Panstrongylus*, while *Candidatus Rohrkolberia* predominates in *Dipetalogaster*.

**Conclusions/Significance:**

The microbiota of triatomine guts represents one of the factors that may interfere with *T. cruzi* transmission and virulence in humans. The knowledge of its composition according to insect species is important for designing measures of biological control for *T. cruzi*. We found that the predominant species of the bacterial microbiota in triatomines form a group of low complexity whose structure differs according to the vector genus.

## Author Summary

Chagas disease is one of the most important endemic diseases of South and Central America. Its causative agent is the protozoan *Trypanosoma cruzi*, which is transmitted to humans by blood-feeding insects known as triatomine bugs. These vectors mainly belong to *Rhodnius*, *Triatoma* and *Panstrongylus* genera of *Reduviidae*. The bacterial communities in the guts of these vectors may have important effects on the biology of *T. cruzi*. For this reason, we analyzed the bacterial diversity hosted in the gut of different species of triatomines using cultivation-independent methods. Among *Rhodnius* sp., we observed similar bacterial communities from specimens obtained from insectaries or sylvatic conditions. Endosymbionts of the *Arsenophonus* genus were preferentially associated with insects of the *Panstrongylus* and *Triatoma* genera, whereas the bacterial genus *Serratia* and *Candidatus Rohrkolberia* were typical of *Rhodnius* and *Dipetalogaster*, respectively. The diversity of the microbiota tended to be the largest in the *Triatoma* genus, with species of both *Arsenophonus* and *Serratia* being detected in *T. infestans*.

## Introduction

Chagas disease [1], which was first described by Chagas [2] as the American human trypanosomiasis, is a potentially life-threatening illness caused by the protozoan parasite *Trypanosoma cruzi*, which is transmitted via obligate hematophagous insect vectors classified within the family *Reduviidae*, subfamily *Triatominae*, commonly known as kissing bugs. Chagas disease is a tropical endemic disease found over large areas of South and Central America and has been ranked as one of the most important diseases in Latin America in terms of social and economic impacts [3], [4]. According to the WHO statistics for 2010, an estimated 10 million people are infected with *T. cruzi* worldwide, mostly in Latin America, with 30% of chronically infected individuals exhibiting cardiac alterations and 10% showing digestive, neurological or mixed alterations. More than 25 million people are at risk of the disease. It is estimated that in 2008, Chagas disease killed more than 10,000 people (http://www.who.int/mediacentre/factsheets/fs340/en/index.html).

In Colombia, the annual treatment costs for chronic Chagas disease patients vary from $46 for a patient with cardiomyopathy without congestive heart failure treated in a basic care facility to approximately $7,900 for a patient with congestive heart failure requiring a specialized level of care. In Mexico, the cost per admitted patient varies from $4,463 to $11,839, while it has been reported to be $3,864 on the average in Brazil. Healthcare costs must be considered together with prevention costs, i.e., a minimum of $30/house. These costs multiplied by the infected population provide an idea of the total costs of Chagas disease for Latin American economies [5]. Despite the continuous pressure for vaccine or drug development, no suitable solution for addressing this situation has been developed thus far. The best strategy for combating Chagas disease is still controlling the triatomine population via insecticide application.

According to their association with humans, it is common to characterize triatomines as domestic, peridomestic or sylvatic. Domestic and peridomestic triatomines create the greatest public concern due to their impact on human populations. The species comprising these groups depend on latitude [6]. Similarly, the type of trypanosome transmitted varies according to geographic localities and the species of insect vectors. Because of co-evolutionary processes, it is often observed that local vector species show a higher rate of infection with local *T. cruzi* strains [7]. Many factors can affect *T. cruzi* gut colonization, including the intestinal microbiota [8], [9]. Thus, the vector may act as a biological filter for the parasites [9], [10]. Based on GMO technology and the coprophagous habits of triatomines, it has been proposed that triatomine resistance of *T. cruzi* gut infection could be increased by natural autoinoculation with paratransgenic symbionts [11].

Different bacterial groups have been found in triatomine guts (see [10]). However, the spectrum of bacterial species identified in laboratory cultures does not necessarily reflect the relative frefquency of these species under natural conditions [10]. Identification of the main bacterial groups in triatomine guts is important, as it may influence the selective pressures on *T. cruzi* [12]. Cultivation-independent methods, such as PCR-DGGE, full-length 16S rDNA sequencing and other molecular approaches, have been widely applied for describing insect gut microbiota and have revealed substantial bacterial diversity as well as groups of uncultivable microbes [13]. These methods offer the advantage of being performed independent of culture medium and providing a quantitative picture of the dominant microbiota present.

In this report, we applied PCR-DGGE and library sequencing approaches to assess the diversity of bacterial communities in the guts of triatomine specimens from insectary colonies and from the field based on 16S rDNA analysis. We show that the microbiota of the triatomine guts are predominately composed of a few bacterial species that tend to be specific to the vector species; i.e., *Arsenophonus* was preferentially associated with vectors of the *Panstrongylus* and *Triatoma* genera, while *Serratia* and *Candidatus Rohrkolberia* were typical of *Rhodnius* and *Dipetalogaster*.

## Materials and Methods

### Insect colonies, field capture and gut dissection

A total of 54 triatomines in the 5^th^ instar larval stage including both males and females belonging to different genera (*Dipetalogaster maximus*, *Panstrongylus megistus*, *Triatoma infestans*, *Triatoma vitticeps*, *Rhodnius prolixus* and *Rhodnius neglectus*) were obtained from insectary colonies maintained over approximately 20 generations at the *Laboratório de Doenças Parasitárias* (Fiocruz, IOC) using chicken as a blood source.

Additionally, nine individuals of *Rhodnius prolixus* in the 5^th^ instar larval stage fed with rabbit blood were obtained from another insect collection maintained as described by Garcia and Azambuja [14], and seven sylvatic 5^th^ instar larvae or adults of *Rhodnius sp.* were directly captured from palm trees (*Attalea maripa*) at Oriximiná, PA, Brazil (Amazon region) as described by Abad-Franch et al. [15] and kept isolated from the other specimens without receiving a blood meal until dissection. Insects that were separated from colonies were dissected 7–10 days after feeding. Dissection of the insects was performed using two fine forceps to open the dorsal side of specimens from the posterior end of the abdomen toward the last thoracic segment. Meticulous dissection of the whole gut was performed using a sterile ultrafine insulin syringe needle. Feces were obtained by abdominal compression or spontaneous dejections immediately after feeding. Guts and feces were collected in sterile Eppendorf tubes and maintained at −20°C until use. All steps were performed under aseptic conditions.

### DNA extraction from insect guts and feces

DNA was extracted from feces and gut samples using the Fast DNA Spin Kit for soil (Qbiogene, BIO 101 Systems, CA, USA) according to the manufacturer's protocol. DNA concentrations were determined using a NanoDrop spectrophotometer (Thermo Fisher Scientific Inc.). The DNA extracts were visualized on 0.8% (w/v) agarose gels to assess their integrity and purity.

### PCR amplification of bacterial 16S rDNA fragments for DGGE

Fragments of 16S rDNA (corresponding to the V6–V8 region of the *E. coli* 16S rDNA gene) were amplified via PCR using the primers 968F-GC (5′-CGC CCG CCG CGC CCC GCG CCC GTC CCG CCG CCC CCG CCC G AAC GCG AAG AAC CTT AC-3′) and 1401R (5′-CGG TGT GTA CAA GAC CC-3′) as described by Nübel et al. [16]. The 50 µl reaction mix contained 1 µl of template DNA (corresponding to approximately 50 ng), 10 mM Tris-HCl (pH 8.3), 10 mM KCl, 2.5 mM MgCl_2_, 0.2 µM of each dNTPs, 1.25 U of *Taq* DNA polymerase (Promega, Madison, WI, U.S.A.), and 0.2 µM of each primer. The amplification conditions were 1×2 min at 94°C followed by 35×1 min at 94°C, 1.5 min at 48°C, and 1.5 min, 72°C, and a final 10 min extension at 72°C. Negative controls (without DNA) were included in all amplifications. The PCR products were analyzed by agarose gel electrophoresis (1.4% gel) and ethidium bromide staining [17]. Amplicons were stored at −20°C until DGGE analysis.

### Denaturing gradient gel electrophoresis (DGGE)

DGGE was carried out as described by da Mota et al. [18] using a Bio-Rad DCode Universal Mutation Detection System (Bio-Rad Laboratories, Munich, Germany). PCR products (15 µl) were applied onto 6% (w/v) polyacrylamide gels in 1× TAE buffer (40 mM Tris-acetate [pH 7.4], 20 mM sodium acetate, 1 mM disodium EDTA) containing a denaturing gradient of urea and formamide varying from 45% to 65%. The gels were run for 15 h at 60°C at 100 V. After electrophoresis, the gels were stained for 30 min with SYBR Green I (Invitrogen - Molecular Probes, SP, Brazil) and photographed under UV light by using a Typhoon Trio apparatus (Amersham Pharmacia Biotech). SYBR Green-stained bands were retrieved by excision from the DGGE gels under UV illumination and eluted in water for sequencing.

### PCR conditions for 16S rDNA cloning

The procedure described by Massol-Deya et al. [19] was used to amplify ∼1.5 Kb fragments of the 16S rDNA gene of each insect specimen via PCR with the universal primer pair pAf (5′-AGA GTT TGA TCC TGG CTC AG-3′) and pHr (5′-AAG GAG GTG ATC CAG CCG CA-3′). The amplification conditions were as follows: 35 cycles of 92°C for 1 min 10 s, 48°C for 30 s and 72°C for 2 min 10 s. A hot start (2 min 10 s at 92°C) was applied to avoid initial mispriming and enhance the specificity of the amplifications. A final extension step was run for 6 min 10 s at 72°C, and the reaction tubes were then cooled to 4°C. A negative control (without DNA) was included in all amplifications. DNA preparation and PCR products were visualized after electrophoresis as described above.

### Cloning and DNA sequencing

Following PCR amplification or band purification with a QIAquick Gel Extraction Kit (Qiagen Inc.), DNA fragments were cloned into the pJET2.1/blunt vector using the CloneJET PCR Cloning Kit according to the instructions of the manufacturer (Fermentas). After transformation of competent *E. coli* DH5α cells, clones were picked, and the presence of inserts of the correct size was assessed via PCR using forward (5′-CGACTCACTATAGGGAGAGCGGC-3′) and reverse (5′-AAGAACATCGATTTTCCATGGCAG-3′) pJET1.2 primers. The clones were sequenced using the same primers in an ABI Prism 3730 automatic sequencer (Applied Biosystems, Foster City, CA, USA). The 101 sequences obtained were deposited in GenBank under the accession numbers JQ410794–JQ410894.

### DGGE band identification

Base calling and low quality sequence trimming from electropherogram files were performed with Phred [20]. The taxonomic position of bacterial genera corresponding to 35 DGGE bands was assessed by comparing their sequences to sequences in GenBank (Rel. 184, June 2011) according to the best BLASTn hits (http://blast.ncbi.nlm.nih.gov/, accessed by 2011-06-08).

### Molecular phylogeny

We first removed plasmid vector sequences with cross-matching and then discarded putative chimeras using the Mallard program [21]. Full-length 16S rDNA sequences were obtained by assembling valid insert sequences with CAP3 [22]. The full-length 16S rDNA sequences and their homologous pairs from GenBank corresponding to the best BLASTn hits were aligned using MUSCLE v3.7 [23]. We constructed maximum likelihood phylogenetic trees with 1,000 bootstrap replicates using the generalized time-reversible (GTR) model [24]. The GTR model is the most general, as it is a neutral, independent, finite-site and time-reversible model. This process allowed accurate inference of the phylogenetic relationships of the full 16S rDNA sequences with their closest relatives of known taxonomic positions.

### Operational taxonomic units and richness estimation

Sequences aligned with MUSCLE v3.7 were formatted according to PHYLIP and used to construct distance matrices for each library with DNADIST (provided in the PHYLIP 3.6 package, [25]) using the default parameters and Jukes-Cantor as the substitution model. The distance matrices were used as input files for MOTHUR v1.14 [26] to define operational taxonomic units (OTU) on the basis of a similarity distance cutoff of 0.03 (OTU_0.03_). Sequences belonging to the same cluster based on reference to OTU_0.03_ were circumscribed with ellipses in the phylogenic trees that we identified using Greek symbols for the purpose of clarity. Although this cutoff distance can be seen as arbitrary, it is often helpful to think of OTUs defined by distances of 0.03 as corresponding to a species [27]. Then, we calculated the Chao1 index [28], which measures the absolute value of species richness. To estimate the relationship between the expected OTU richness and the sampling depth, we used rarefaction curve methodology [29], [30]. Good's coverage estimator [31] was used to calculate the sample representativeness with the formula C = 1−(ni/N)×100 [28], where N is the total number of clones analyzed, and n_i_ is the number of clones that occurred only once among the total number of clones analyzed using OTU_0.03_ [32]

## Results

**Figure 1:**
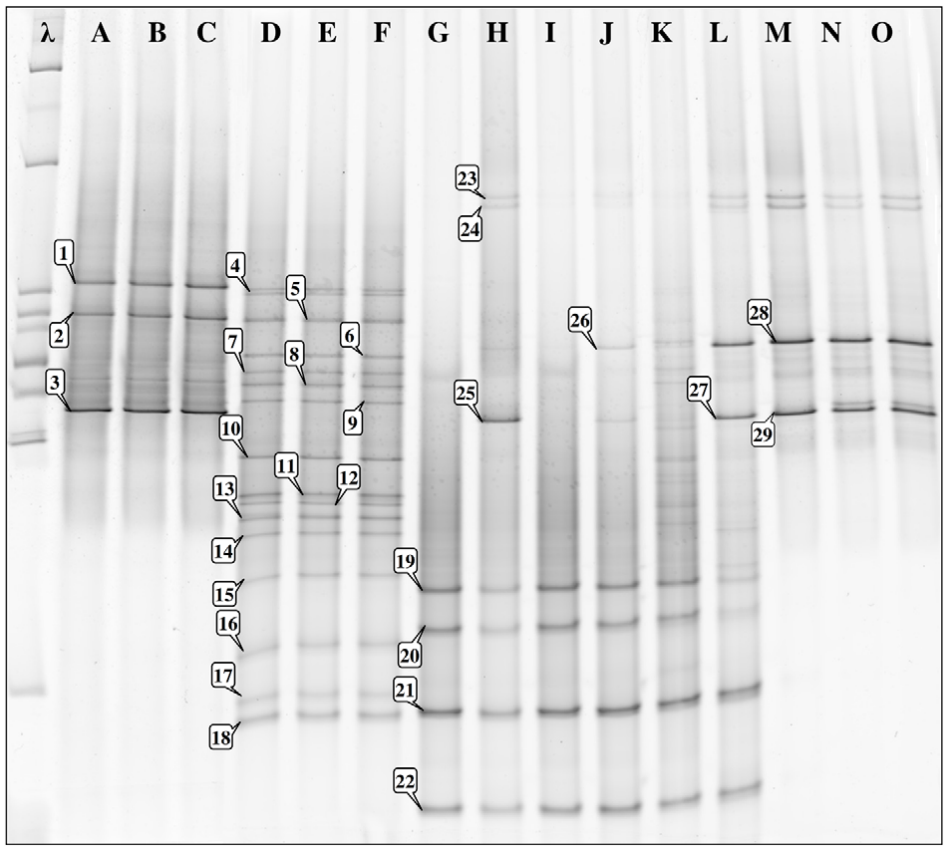
DGGE fingerprints of bacterial 16S rDNA gene fragments amplified from gut microbiota. The λ lane contains 5 µl of a BenchTop 1 Kb DNA ladder (Promega). The other lanes are for *Dipetalogaster maximus* (A,B,C), *Panstrongylus megistus* (D,E,F), *Triatoma infestans* (G,H,I), *Triatoma vitticeps* (J,K,L), *Rhodnius neglectus* (M,N and O). Band identification corresponds to *Candidatus Rohrkolberia/Pectobacterium* (1 to 3), *Arsenophonus* (4 to 22), and *Serratia* (23 to 29).

DGGE is a simple method that is well suited to characterize the global complexity of bacterial communities, such as found in triatomine guts from insectary colonies or field specimens. When analyzing the electrophoresis migration patterns of 16S amplicons from guts of *Dipetalogaster maximus* (Fig. 1, lanes A–C), *Panstrongylus megistus* (Fig. 1, lanes D–F), *Triatoma infestans* (Fig. 1, lanes G–I), *Triatoma vitticeps* (Fig. 1, lanes J–L) and *Rhodnius neglectus* (Fig. 1, lanes M–O) using this system, we observed band patterns that are characteristic of these species and were conserved among triplicate specimens of the same vector species.

**Figure 2:**
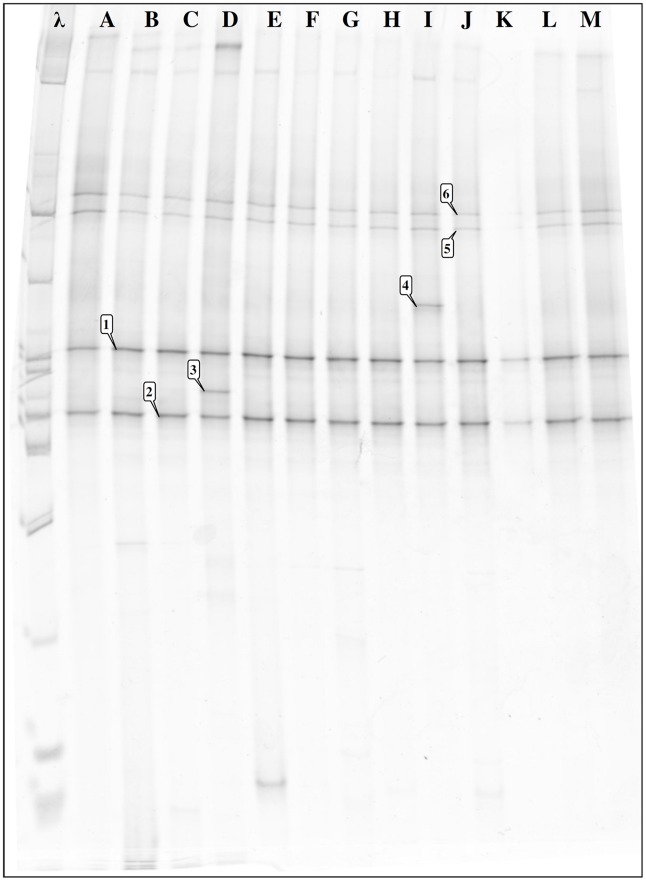
DGGE fingerprints of bacterial 16S rDNA gene fragments amplified from *Rhodnius*. The λ lane contains 5 µl of a BenchTop 1 Kb DNA ladder (Promega). The other lanes are for *R. prolixus* fed with rabbit blood (A); *R. prolixus* fed with chicken blood (B); *R. neglectus* fed with chicken blood (C); guts of Amazon sylvatic *Rhodnius sp.* adults (D, E, F, G); 5th instar *larvae* (H, I, J); and feces (K), (L, other sylvatic feces pool) and (M, insectary feces pool). Band identification matches *Serratia* (1, 2, 5 and 6), *Rohrkolberia* (3) and *Wolbachia* (4).

Further characterization of these bands by DNA sequencing revealed that their corresponding bacterial species were essentially members of the *Enterobacteriaceae*, particularly of the three genera *Candidatus Rohrkolberia* (bands 1–3), *Arsenophonus* (bands 4–22) and *Serratia* (bands 23–29). Some bands (23–25) related to *Serratia* were shared by different insect genera, i.e., *Triatoma* and *Rhodnius*. Additionally, *Arsenophonus* sequences were found in *Panstrongylus* (bands 4–18) and *Triatoma* (bands 19–22), but not in *Rhodnius* or *Dipetalogaster*. The fingerprints of *Triatoma infestans* and *Triatoma vitticeps* were similar, suggesting that the main species in the bacterial communities in triatomine guts are specific to the genus of their hosts. When the DGGE fingerprints of wild specimens of *Rhodnius* sp. collected from the Amazon (Fig. 2, lanes D–L) were compared to those of specimens of *Rhodnius prolixus* (Fig. 2, lanes A–B) and *Rhodnius neglectus* (Fig. 2, lane C) from insect collections grown under captivity and fed with rabbit or chicken blood, we actually observed similar profiles (Fig. 1, bands 28 and 29; Fig. 2, bands 1 and 2), although some specific bands (Fig. 2, bands 3 and 4) could be only observed in the sylvatic specimens. The profiles were similar whether guts (Fig. 2, lanes D–J) or only feces (Fig. 2, lanes L–M) were analyzed, showing that these bacteria (Fig. 2, bands 1,2,5 and 6) are most probably free in the *Rhodnius* gut lumen. The profiles were also similar in insects at the 5^th^ instar larval (Fig. 2, lanes H–J) and adult stages (Fig. 2, lanes D–G). The sequences of the specific bands could be associated to *Candidatus Rohrkolberia cinguli* (GenBank: FR729479.1) or *Erwinia chrysanthemi* strain NZEC151 (GenBank: EF530551.1) (Fig. 2, band 3) and *Wolbachia* (Fig. 2, band 4), which is an endosymbiont that infects a wide range of insect hosts (20 to 75% of insect species, see refs in [33]), such as *Microcerotermes* sp. (GenBank: AJ292347.1), *Pseudolynchia canariensis* (GenBank: DQ115537.1) and *Supella longipalpa* (GenBank: FJ152101.1).

The full-length 16S rDNA sequences (1.5 kb) are larger than the partial sequences of 16S bands separated by DGGE (approximately 430 pb). Therefore, the full-length sequences allow classification of bacteria at the species level rather than just the genus level. Thus, full-length 16S rDNA sequences obtained from libraries of 16S rDNA clones allowed the description of the bacterial community structure in the investigated specimens in more details.

**Figure 3:**
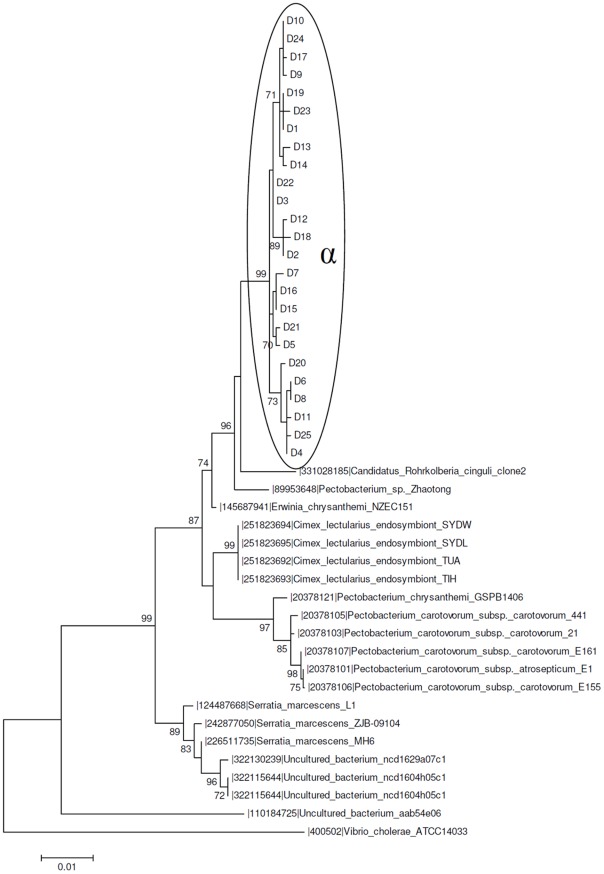
Maximum likelihood phylogenetic tree of a 16S rDNA library of *D. maximus* (Dm) gut microbiota. The 25 sequences (D in the phylogenetic tree) were obtained from insectary individuals (FIOCRUZ/IOC) and compared with GeneBank sequences. The percent values on branches are based on 1,000 bootstrap replicates. The α ellipse represents an OTU_0.03_ cluster obtained with MOTHUR that is shared by *D. maximus* and *R. polixus*.

The simplest microbiota structure was observed in *D. maximus* (Fig. 3), which presented only one OTU_0.03_ cluster (D1–25), designated α, including all sequences obtained from its rDNA library.

**Figure 4:**
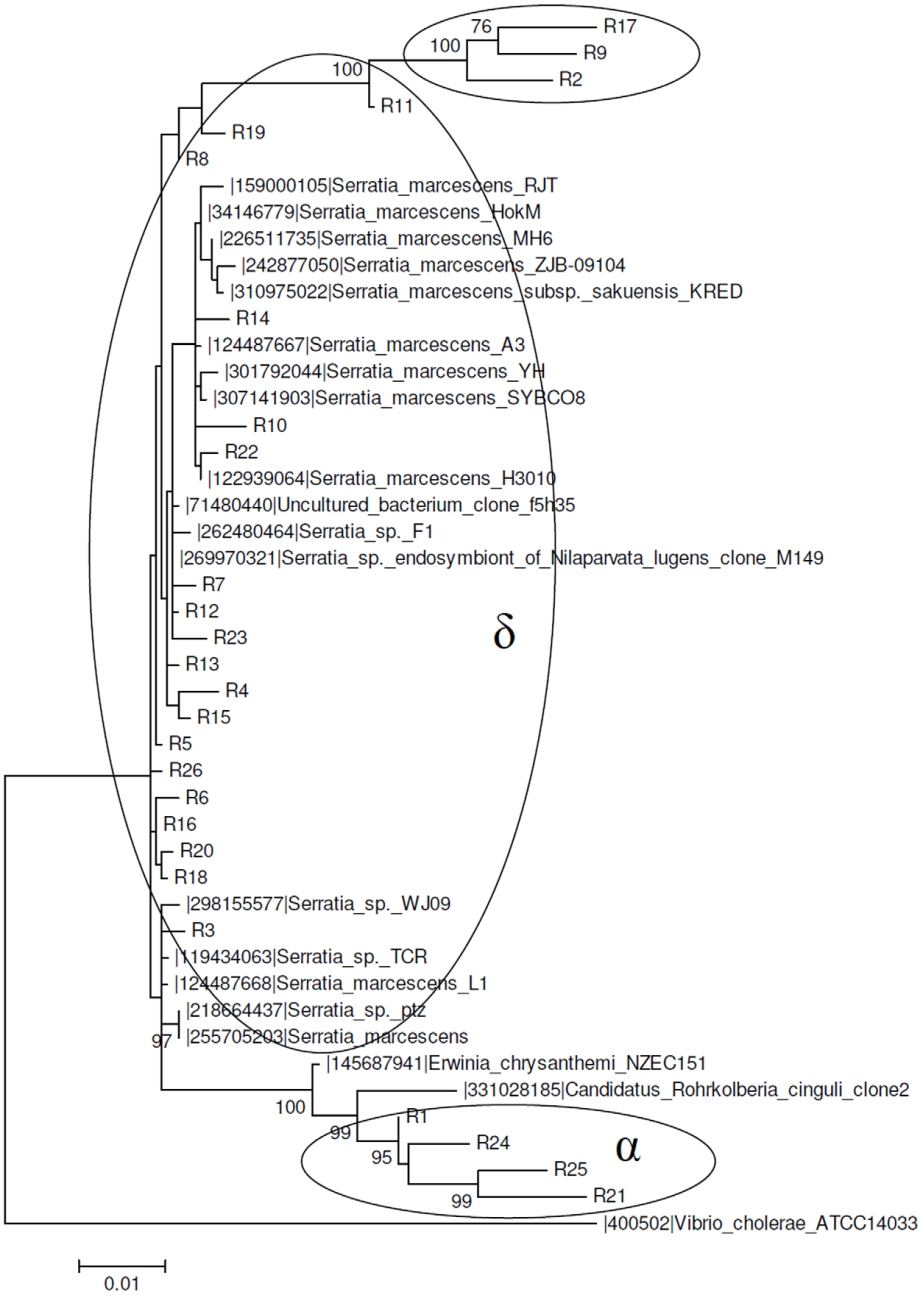
Maximum likelihood phylogenetic tree of a 16S rDNA library of *R. polixus* (Rp) gut microbiota. The 26 sequences (R in the phylogenetic tree) were obtained from insectary individuals (FIOCRUZ/IOC) and compared with GeneBank sequences. The percent values on branches are based on 1,000 bootstrap replicates. The ellipses represent OTU_0.03_ clusters obtained with MOTHUR. The α ellipse represents an OTU_0.03_ cluster shared by *D. maximus* and *R. polixus*. The δ ellipse represents an OTU_0.03_ cluster shared by *R. polixus* and *T. infestans*.

Sequences belonging to this α OTU_0.03_ cluster were also observed in the *R. prolixus* microbiota (Fig. 4). The sequences from the α cluster were grouped along the same phylogenetic branch and were closely related to *Candidatus Rohrkolberia cinguli* (DQ418491.1). One of the sylvatic specimens of *Rhodnius sp.* also showed a DGGE band (Fig. 2, lane D, band 3) related to *Candidatus Rohrkolberia cinguli*. *Candidatus Rohrkolberia cinguli* is a new genus and species name recently proposed for the newly characterized clade of obligate intracellular symbiotic bacteria found in the midgut epithelium of the bulrush bug *Chilacis typhae* [34]. In *R. prolixus*, we found two additional OTU_0.03_ clusters representing approximately 85% of the bacterial microbiota related to *Serratia marcescens* strains (Fig. 4). Cluster δ was the major cluster and represented approximately 73% of the sequences found in the *R. prolixus* library. The minor cluster (R2, R9, R17) is most likely related to *Serratia* sp.

**Figure 5:**
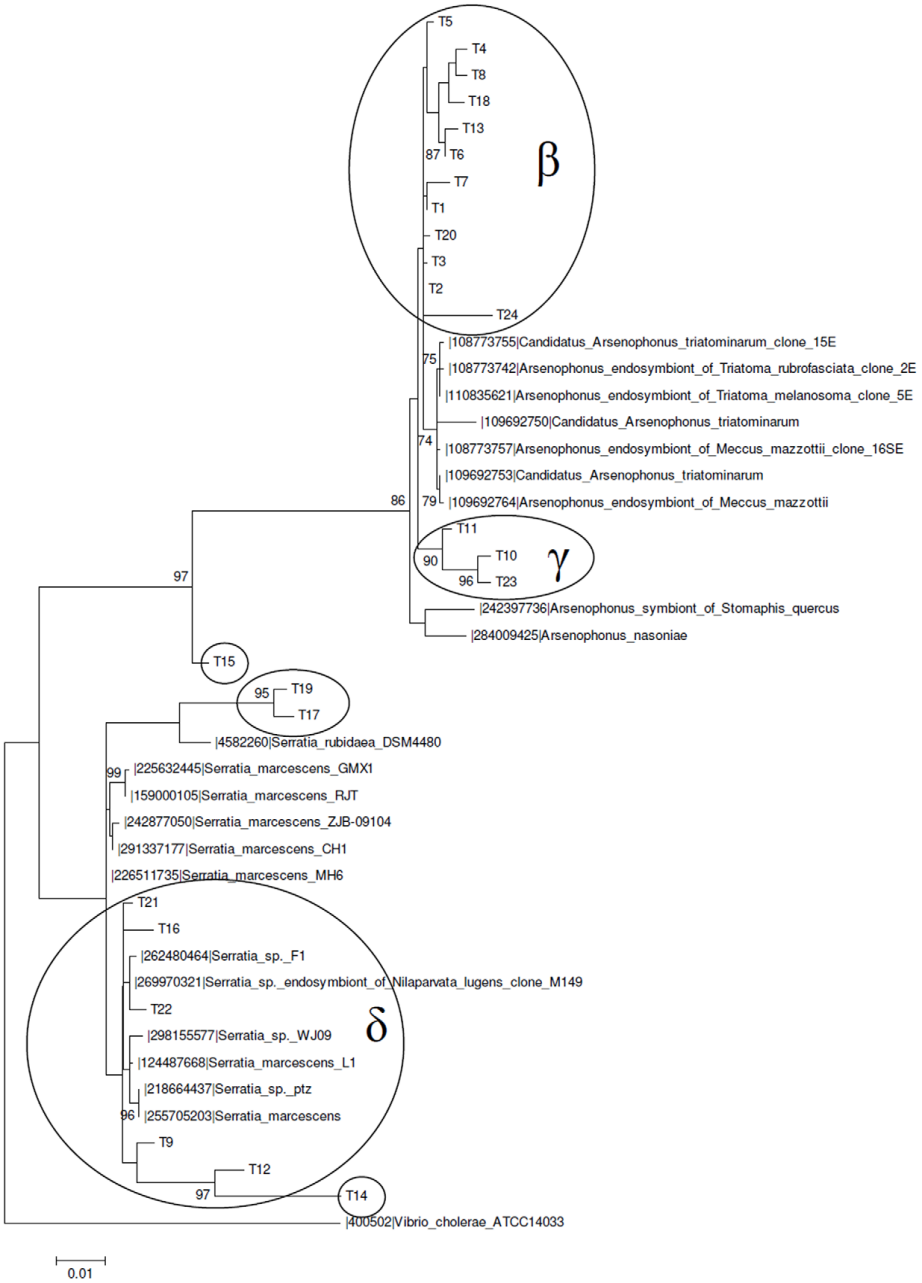
Maximum likelihood phylogenetic tree of a 16S rDNA library of *T. infestans* (Ti) gut microbiota. The 24 sequences (T in the phylogenetic tree) were obtained from insectary individuals (FIOCRUZ/IOC) and compared with GeneBank sequences. The percent values on branches are based on 1,000 bootstrap replicates. The ellipses represent OTU_0.03_ clusters obtained with MOTHUR. The γ and β ellipses represent OTUs_0.03_ clusters shared by *T. infestans* and *P. megistus*. The δ ellipse represents an OTU_0.03_ cluster shared by *T. infestans* and *R. polixus*.

Sequences similar to the δ OTU_0.03_ cluster were also observed in the *T. infestans* microbiota (Fig. 5). Among the triatomine vectors that we investigated here, *T. infestans* presented the most complex microbiota structure, with six OTUs_0.03_ clusters. In addition to the δ cluster, we found (i) an OTU_0.03_ represented by only one sequence (T14) closely related to the δ cluster, (ii) an additional uncharacterized singleton OTU_0.03_ represented by T15, (iii) an OTU_0.03_ including two sequences (T17, T19) related to *Serratia rubidaea* and (iv) two clusters (β and γ) associated with the *Arsenophonus* endosymbionts, representing 62% of the whole set of sequences.

**Figure 6:**
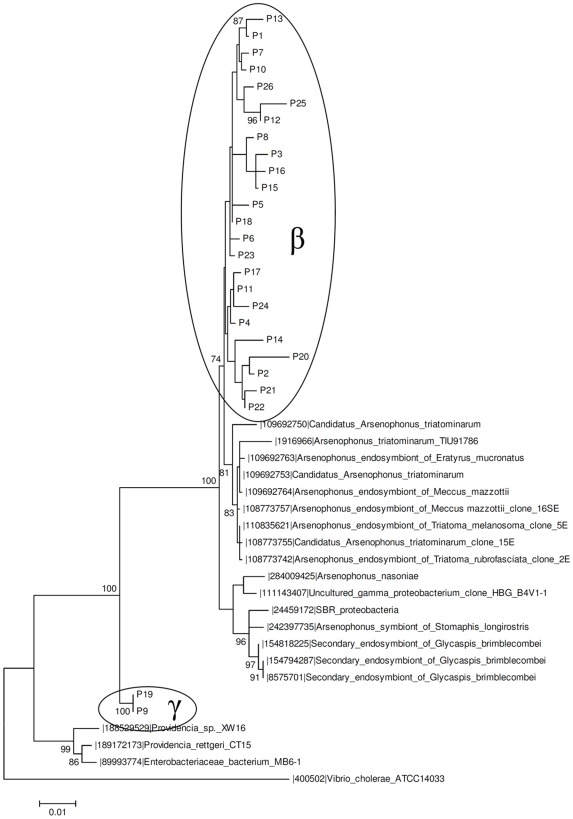
Maximum likelihood phylogenetic tree of a 16S rDNA library of *P. megistus* (Pm) gut microbiota. The 26 sequences (P in the phylogenetic tree) were obtained from insectary individuals (FIOCRUZ/IOC) and compared with GeneBank sequences. The percent values on branches are based on 1,000 bootstrap replicates. The ellipses represent OTU_0.03_ clusters obtained with MOTHUR. The γ and β ellipses represent OTUs_0.03_ clusters shared by *P. megistus* and *T. infestans*.

Sequences similar to clusters β (P1–8, P10–18, P20–25) and γ (P9, P19) from *T. infestans* were also observed in *P. megistus* (Fig. 6), where they represented 92% of the whole sequence set.

**Figure 7:**
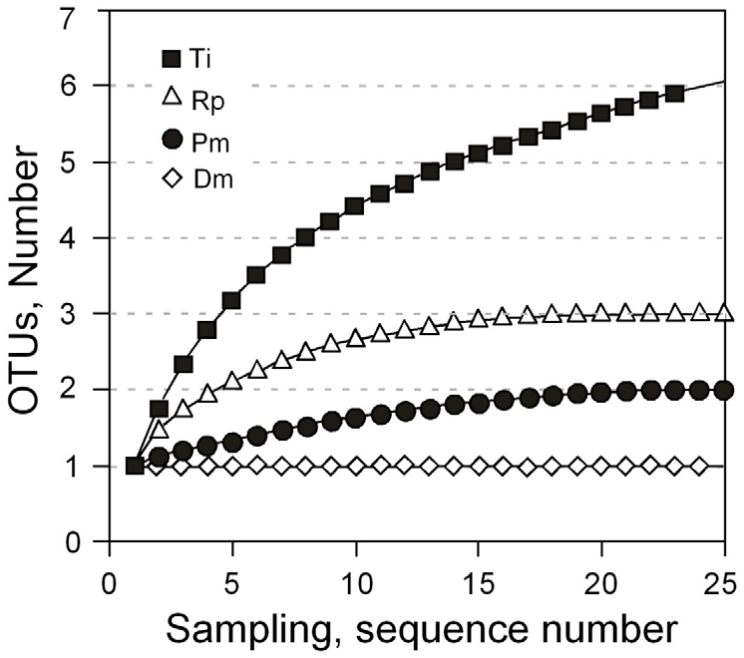
Rarefaction curves of 16S rDNA sequences from triatomine gut microbiota. The rarefaction curves were calculated using MOTHUR OTU_0.03_ in libraries of *P. megistus* (Pm), *T. infestans* (Ti), *R. prolixus* (Rp) and *D. maximus* (Dm). The plot shows the number of new bacterial species as a function of the number of clones sequenced.

To better characterize the relative bacterial species richness among the triatomines, we used the Chao index. The Ti library exhibited the highest species richness, with 6.5 expected OTUs_0.03_ (confidence interval: 6.03–14.30). The *R. prolixus* library showed 3 OTUs_0.03_, while the *P. megistus* library showed 2 OTUs_0.03_, and *D. maximus* showed 1 OTU_0.03_ according to the Chao index. There was no confidence interval associated with *R. prolixus*, *P. megistus* and *D. maximus* because there are no unseen species expected in these triatomine species given the rarefaction curve saturation (Fig. 7). The trend of the rarefaction curves suggests that the bacterial composition spectrum observed in the *T. infestans* library could indeed be slightly larger than reported here (Fig. 7). The Good's coverage estimator calculated from the 16S rDNA sequences of *R. prolixus*, *P. megistus* and *D. maximus* libraries was 100%, demonstrating the representativeness of our results compared to real conditions. In comparison, the same index showed that our sequencing coverage of the *T. infestans* library was 91.6%.

## Discussion

A low level of microbiota complexity seems to be frequent in insect guts [34]–[38], except in particular cases such as termites [39]. This study shows that the triatomine gut microbiota, as revealed by DGGE, are composed of a few predominant bacterial species that ultimately differ according to the insect vector. Interestingly, the banding patterns were conserved among specimens of the same species, suggesting that the bacterial communities in triatomines are well adapted to their hosts. In addition, we found the same spectrum of bacterial species when using different primers pairs for DGGE and full-length 16S rDNA sequencing, which reduces the probability of a bias at the level of PCR amplification. Blood is of course sterile, and the sucking mouth parts of triatomines are adapted to blood consumption directly from vertebrate host capillaries, minimizing contamination by skin penetration. However, triatomines have multiple opportunities to inoculate themselves with bacteria, as they are coprophagous [40], [41].

The habit of feces consumption by triatomines may explain the similarity between the DGGE fingerprints of the gut microbiota of individual insects within the same genus. It also suggests that the bacterial communities found within these vector species are rather stable and well adapted to their environment. Given the potentially large spectrum of bacterial species in triatomine feces due to environmental contamination, it must be concluded that the low number of prevalent bacterial populations in triatomine guts despite their coprophagous behavior is due to regulation by the host vector [42]. Several humoral [38], [42], [43] and cellular mechanisms involved in vector defenses have in fact been described. These mechanisms mainly include (i) antimicrobial peptides that could restrict the bacterial diversity in the gut, such as *prolixicin* produced by *Rhodnius prolixus* [44], (ii) lysozyme activity, (iii) prophenoloxidase activation, (iv) phagocytosis and hemocyte microaggregation, (v) nitric oxide and superoxide production and (vi) trypanolytic proteins (see refs in [10]). Moreover, the microbiota also participates in other basic functions, such as digestion and vitamin production [45]. It is the specific biochemical balance between host and microbiota factors that determines the features of the environment where a parasite has to adapt, and these features are expected to interact with its virulence [10]. The detection limit of bacterial DNA among eukaryotic DNA via PCR under normal conditions has been reported to be between approximately 4×10^2^ and 4×10^3^ fragments [37]. As a consequence, we believe that our PCR results simply reflect the most frequent bacterial species, which does not exclude potential presence of other species in minute amounts. In addition, some weak bands from the DGGE gels were not characterized, and the possibility cannot be excluded that some taxa escaped our analyses. However, we also showed that more than 90% of the information related to the gut microbiota of the triatomine specimens in our investigation has been considered here via analysis of rarefaction curves obtained for 16S rDNA libraries.

One advantage of DGGE analysis is the possibility of extracting bands from the gels followed by performing sequence analysis of the purified amplicons and identifying community members belonging to different phyla, such as Actinobacteria, Proteobacteria, Firmicutes, and Deferribacteres, by comparison to reference databases [18], [37], [46]. Investigation of the gut microbiota of different triatomine species using bacterial culture methods revealed a bacterial community of limited diversity characterized by several species of *Enterobacteriaceae* [10], [40], with some of them being eventually pathogenic to humans. In our analyses, we mainly detected members of the *Enterobacteriaceae* (*Serratia, Candidatus Rohrkolberia/Pectobacterium* and *Arsenophonus*), which suggests that this group is predominant in the guts of triatomines. *Enterobacteriaceae* appear to be frequent in insects, particularly in insect vectors whose diets are limited to a few food sources [35], [45], [47].

In their review of microbiota complexity, Vallejo et al. [10] reported 6, 25 and 26 bacterial species in *T. infestans*, *R. prolixus* and *P. megistus*, respectively. However, these numbers were obtained by different authors across very different conditions and under *in vitro* culture. It is clear that this list does not show the relative abundance of these species in the triatomine gut. In contrast, the present study provides an indication of the predominant species of the bacterial microbiota across triatomine species in insectaries and field conditions, including obligatory intracellular symbionts that cannot be detected using traditional culture methods [34].

Many complex molecules produced by hosts or microbiota are present in the insect gut lumen, such as hydrolytic enzymes, peptides, vitamins, cofactors and antimicrobial factors. These molecules can stimulate some bacteria, but they can inhibit the growth of many other competing members in this environment. For these reasons and many others, it has been recognized that the *in vitro* conditions of bacteria culture on artificial media do not mimic those of the natural conditions in insect guts [13].

Diagnosis of the number of predominant bacterial species is a quantitative concept when it involves 16S rDNA, which deserves some comment. Because variation is continuous among sequences, criteria based on genetic distance must be applied to discriminate among species, which is the reason that we applied the cutoff of 0.03 to discriminate among OTUs. Although this cutoff distance can be seen as arbitrary, it is often helpful to think of OTUs that are defined by a distance of 0.03 as corresponding to a species, of 0.05 as corresponding to a genus, of 0.15 as corresponding to a class, and of 0.20 to 0.30 as corresponding to a phylum [27]. The concept of species among bacteria is a difficult issue. At first glance, the large sequence number per cluster may surprise, but deeper observation shows that the genetic diversity between these sequences is equivalent to that among strains of the same bacterial species, as found in GenBank. This is particularly obvious for *Serratia* in cluster δ. We found relatively good agreement between the description of microbiota complexity according to (i) OTU_0.03_ criteria [27], (ii) branch numbers in phylogenetic trees, and (iii) the species richness index of Chao.

The number of dominant bacterial OTUs_0.03_ clusters in *T. infestans* was ∼6 according to the Chao index, with an upper limit of approximately 14 according to the inference of unseen species. This index has the advantage of presenting clear and rigorous non-parametric statistics [28]. Based on this observation, we can conclude that the microbiota diversity according to the prevalent bacterial OTUs_0.03_ is at least two times greater in *T. infestans* compared to the other vector species, suggesting a different relationship between microbiota and vector factors with possible consequences for protozoan parasitism.

A methodology similar to that used in this study, including DNA extraction, universal primers and PCR-DGGE, allowed successful recovery of 16S rDNA sequences related to *Rhodococcus* sp. (GU585554), *Gordonia* sp. (GU585556, GU585557) and other Actinomycetales from different environments [18], [48]. Although sequences belonging to *Rhodococcus* were not recovered in the present study, we cannot exclude the association of weak DGGE bands with *Rhodococcus* [40].

In *Rhodnius* specimens, *S. marcescens* was the predominant species observed; this species is a free-living bacterium that produces a red pigment known as *prodigiosine*, which has recently received renewed attention due to its reported antibacterial, antifungal, antiprotozoan [49], immunosuppressive and anticancer properties [50]. Moreover, *Serratia marcescens* has been reported to utilize the type VI secretion system (T6SS) to target bacterial competitors [51]. It was demonstrated that T6SS exhibits dramatic antibacterial killing activity against several other bacterial species [52], [53], favoring *S. marcescens* strains in *Drosophila* [51]. The *S. marcescens* found in *Triatoma* sp. and *Rhodnius* sp. guts could reduce the number and diversity of other extracellular bacteria in the gut lumen via the same process, as the antibacterial activity of *S. marcescens* T6SS appears to act through direct bacterium-to-bacterium contact [51]. In addition, *S. marcescens* can also lyse *T. cruzi* through the action of D-mannose fimbriae, which adheres to the parasite surface [54].

The genus *Arsenophonus* represents a group of endosymbiotic, mainly insect-associated bacteria with a broad spectrum of insect and even plant hosts [55]. *Arsenophonus* includes lineages with a rapidly increasing number of closely related symbionts reported from phylogenetically distant hosts [55], [56]. A member of this genus, *Candidatus Arsenophonus triatominarum*, has been isolated from *T. infestans* [56]. This bacterium is an intracellular endosymbiont found in hemolymph, heart tissue, salivary glands, neural ganglia, visceral muscles, nephrocytes, ovaries, testes, and dorsal vessels that lives in the cytoplasm of host cells and displays pleiomorphy, with forms ranging from spherical to highly filamentous. In contrast to *Arsenophonus nasoniae* (a symbiont of parasitoid wasps of the genus *Nasonia*), *A. triatominarum* does not grow on artificial culture media but does grow well on *Aedes albopictus* cell lines, which demonstrates that it must be considered to be a P-symbiont [55], and the two species form a distinct lineage of bacteria within the family *Enterobacteriaceae* [56]. The draft sequence of the complete genome of *A. nasoniae* [57], [58] shows that it carries putative hemolysins, alkaline metalloproteases, serralysin, (an insecticidal toxin of *Serratia*) and several other pseudogenized toxin genes that most likely indicate past parasitic activity typical of *Enterobacteriaceae* [58]. In addition, it also carries 8–10 copies of the rDNA operon [57]. Therefore, although we detected several *Arsenophonus* bands by DGGE in *P. megistus*, we attributed this to paralogy rather than orthology. Analysis of these sequences actually revealed a high level of similarity among them, and the Chao1 species richness estimator indicated the presence of only two OTUs in the library, which confirms paralogy, as discussed by Nováková et al. [55]. Moreover, the *Arsenophonus* OTUs from the *P. megistus* and *T. infestans* libraries showed a high level of similarity to the *Arsenophonus* accession in GenBank previously isolated from *Triatoma melanosoma* [59].

When *Candidatus Rohrkolberia cinguli* was reported for the first time in *Chilacis typhae*, a 1.5 kb segment of the eubacterial 16S rRNA gene was amplified by PCR from DNA samples from the midgut epithelium of the hemipteran species *Chilacis typhae* (52 individuals were used for PCR), then cloned and typed by RFLP. All RFLP types of 40 clones were identical. Furthermore, when a 1.65 kb segment of the gammaproteobacteria *groEL* gene was amplified, cloned and sequenced, the RFLP types and sequences of the clones were all the same [34], indicating that the *Rohrkolberia* population is prevalent and well adapted to the host.

Some sequences belonging to the same *Rohrkolberia* OTU cluster in *D. maxima* were also found in *R. prolixus*, demonstrating that *Candidatus Rohrkolberia* is not restricted to one insect genus. In fact, to our knowledge, this is the first time that *Candidatus Rohrkolberia* has been reported in triatomine guts, and this species, together with *Arsenophonus* and *Wolbachia*, the other symbionts found in this study, deserve more attention with respect to paratransgenic strategies.

Interestingly, *Wolbachia*, which is a symbiont that can be transmitted together with *Arsenophonus* [57] by parasitoid wasps of the genus *Nasonia* [56], has been found in a *Rhodnius* specimen from the Amazon. *Wolbachia* has been described in several organs and feces of *Rhodnius pallescens* by Espino et al. [33]. These symbionts belong to α-Proteobacteria and can cause postzygotic reproductive incompatibilities in insects, as they display a tropism for the reproductive tissues of their hosts and are transmitted vertically from insect to insect through ovules or horizontally through parasitoids. Despite the fact that infected insects do not show pathological signs, the presence of *Wolbachia* can result in diverse reproductive alterations in their hosts, including parthenogenesis, feminization, male killing and unidirectional or bidirectional cytoplasmic incompatibility. The relationship between *Wolbachia* and their arthropod hosts ranges from mutualistic to parasitic depending on the *Wolbachia* strain and arthropod species [33]. Introduction of a *Wolbachia* wMel strain isolated from *Drosophila melanogaster* to an adult vector of *Aedes aegypti* allowed successful suppression of dengue transmission in two natural populations of *A. aegypti* within only a few months [60]–[62].

The reduced number of sylvatic samples analyzed in the present study does not permit us to reach a definitive conclusion regarding variations in microbiota between insectary and sylvatic individuals of *Rhodnius*. However, some differences were observed between the prevalent populations of bacteria, including *Wolbachia*, although only seven individuals of sylvatic *Rhodnius* sp. were analyzed.

If DGGE profiles show that the complexity of predominant bacterial species of the *Rhodnius* gut microbiota is apparently low, exhaustive cloning and sequencing will be necessary to reveal bacterial species with a relative minor number in the microbiota analyzed. The present pilot investigation should be used as a basis for future analyses with other specific focuses that would require a more refined approach, such as high-throughput sequencing.

*Rhodnius* has long served as an important physiological laboratory model. Since Wigglesworth's pioneering work [63] on molting and reproduction, a large body of knowledge has been accumulated worldwide. Ultimately, it has been proposed that protozoan parasites inside the guts of vectors should be killed by infecting triatomines under natural conditions with paratransgenic bacteria that are able to produce antimicrobial factors [11], [64], [65]. However, this strategy, known as paratransgenesis [64], should take into consideration potential interactions with the intestinal microbiota under natural conditions. As shown above, the composition of the gut microbiota varies according to the species of insect vector and includes *Serratia*, *Arsenophonus*, *Rohrkolberia* or *Wolbachia* populations, which could affect the success of paratransgenic approaches over time and, thus, long-term host protection. Possible degradation or bioaccumulation of paratransgenic factors by natural microbiota is another matter of concern. Finally, the long-term effect of paratransgenic factors on the mechanisms through which symbiotic microbes can influence the ability of their host to transmit pathogens may be questioned [38], [42], [43].
